# Chronic kidney disease and cognitive performance: NHANES 2011–2014

**DOI:** 10.1186/s12877-024-04917-2

**Published:** 2024-04-18

**Authors:** Te Li, Zhiling Hu, Luyao Qiao, Yao Wu, Ting Ye

**Affiliations:** 1 Department of Nephrology, Nanchang People’s Hospital, Nanchang, Jiangxi, , China; 2 Department of Cardiology, Nanchang People’s Hospital, Nanchang, Jiangxi, , China; 3 Department of Neurology, The Second Affiliated Hospital of Nanchang University, Nanchang, Jiangxi, , China

**Keywords:** Chronic kidney disease, eGFR, Cognitive performance, Cognitive impairment, NHANES, Older adults

## Abstract

**Purpose:**

Previous studies suggest an association between chronic kidney disease (CKD) and cognitive impairment. The purpose of this study was to explore the association between the diverse stages of CKD and the cognitive performance of elderly American adults.

**Methods:**

Data from the National Health and Nutrition Examination Survey (NHANES) 2011–2014 were used. Multivariate adjusted logistic regression, subgroup analysis, and the restricted cubic spline model were used to assess the associations of CKD stage and estimated glomerular filtration rate (eGFR) with cognitive performance. The measures used to evaluate cognitive function included the Consortium to Establish a Registry for Alzheimer’s Disease (CERAD) test, the Animal Fluency test, and the Digit Symbol Substitution test (DSST).

**Results:**

This study included 2234 participants aged ≥ 60 years. According to the fully adjusted model, stages 3–5 CKD were significantly associated with the CERAD test score (OR = 0.70, 95% CI [0.51, 0.97], *p* = 0.033), the Animal Fluency test score (OR = 0.64, 95% CI [0.48, 0.85], *p* = 0.005), and the DSST score (OR = 0.60, 95% CI [0.41, 0.88], *p* = 0.013). In addition, the incidence of poor cognitive function increased with decreasing eGFR, especially for individuals with low and moderate eGFRs. Both the DSST score (*p* nonlinearity < 0.0001) and the Animal Fluency test score (*p* nonlinearity = 0.0001) had nonlinear dose–response relationships with the eGFR. However, a linear relationship was shown between the eGFR and CERAD test score (*p* nonlinearity = 0.073).

**Conclusions:**

CKD, especially stages3–5 CKD, was significantly associated with poor cognitive performance in terms of executive function, learning, processing speed, concentration, and working memory ability. All adults with CKD should be screened for cognitive impairment.

## Introduction

Chronic kidney disease (CKD) and cognitive impairment are common conditions most notably for the aging population and have increasing public health significance [1]. With increased longevity, older adults of American age are at a significant risk of having multiple chronic diseases (such as CKD) and associated functional impairment (such as cognitive impairment). It is estimated that more than one in seven U.S. adults (approximately 35.5 million people; 14% of U.S. adults) have CKD [2, 3]. Patients with CKD have an increased risk of stroke and a high burden of cardiovascular risk factors such as diabetes and hypertension, which in turn are closely associated with cognitive impairment.

Cognitive impairment refers to a deficit in several crucial brain functions, such as memory, learning, attention, and decision-making ability. Cognitive impairment can range from mild to severe, and serious impairment affecting daily living and independence is commonly referred to as dementia [4]. In the U.S., of those at least 65 years of age, there were an estimated nearly 7 million adults with dementia in 2014, and nearly 14 million were projected to be diagnosed by 2060 [5]. Traditional cardiovascular risk factors including diabetes, hypertension, and smoking, are linked to a 20–40% increased risk for dementia in the general population [6]. However, CKD patients face a substantially greater risk of cognitive impairment than the general population. The possible reasons are that CKD patients have a greater risk of atherosclerosis, stroke, and traditional cardiovascular risk factors [7, 8]. Additionally, impaired uremia metabolite clearance, polypharmacy, depression, sleep disorders, and renal anemia could also explain these findings [9, 10].

The association between CKD and cognitive performance is still being studied extensively. On the one hand, some evidence has demonstrated that a decreased estimated glomerular filtration rate (eGFR) in patients with CKD may increase the risk of cognitive impairment [11]. However, these studies were performed in cohorts with comorbidities and limited ethnic diversity and included few participants with advanced CKD. On the other hand, CKD was not as a covariate in large-scale epidemiological studies of cognitive impairment and cardiovascular risk factors [12, 13]. Thus, these findings cannot be generalized to approximately 7.4 million Americans with CKD. As a result, we investigated the relationship between different stages of CKD and cognitive performance in American elderly individuals.

## Methods

### Data collection

**Fig. 1:**
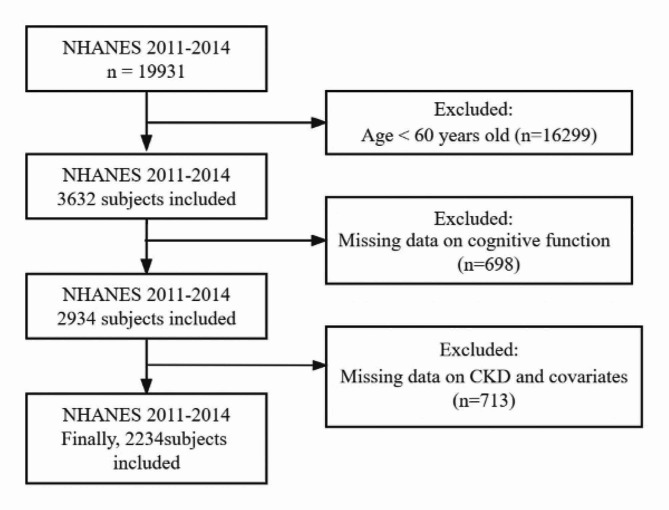
Flowchart of participant selection

The National Health and Nutrition Examination Survey (NHANES) is an ongoing cross-sectional survey whose major purpose is to estimate the number and proportion of noninstitutionalized American civilians with specific diseases and risk factors. Among a total of 19,931 participants in the NHANES 2011–2014, cognitive performance was evaluated in participants aged ≥ 60 years. There were 2934 participants who had valid information on cognitive function were included. Patients with incomplete data about CKD and covariates were excluded, and we included 2234 participants in the final analysis (Fig. 1). All the data were obtained at https://wwwn.cdc.gov/nchs/nhanes/default.aspx.

### Outcome measurement

Cognitive function was measured either during the household interview or as a component of the mobile examination center. A series of tests were used to evaluate cognitive function, including word learning and recall modules from the Consortium to Establish a Registry for Alzheimer’s Disease (CERAD), the Animal Fluency test, and the Digit Symbol Substitution test (DSST). The CERAD test instantly examines delayed recall of new verbal information (memory subdomain), which consists of three consecutive learning trials and a delayed recall [14]. For the learning trials, participants are presented to read aloud ten unrelated words, one at a time. Then participants recalled as many words as possible at the end of presentation of the words. The order of 10 words is changed during the each of three learning trials. Followed the other two cognitive exercises (the Animal Fluency test and DSST), the delayed word recall was completed. The maximum score on each trial was 10, and the total score on the CERAD test was the sum of three learning trials and a delayed recall trial. The Animal Fluency test evaluates categorical verbal fluency, which is a component of executive function [15]. Scores have been shown to assess the severity of cognitive decline [16]. The test does not entirely rely on formal culture-specific educational backgrounds and experiences, but does require the presence of awareness (such as naming animals) [17]. Participants were requested to name as many animals as possible in one minute. Although no upper limit exists, practically, the Animal Fluency test score ranges from 3 to 39. The DSST is a performance module from the Wechsler Adult Intelligence Scale that is used to evaluate processing speed, sustained attention, and working memory ability [18]. The test was conducted using a paper form that has a key at the top containing 9 numbers paired with symbols. Participants had 2 minutes to copy the corresponding symbols in the 133 boxes that adjoin the numbers. The score is the total number of correct matches, and the maximum of score is 105 in this test. In epidemiologic studies with diverse racial and cultural communities, these tests have been frequently used [19–21]. Currently, there are no gold standard cut-off values for determining poor cognitive function according to these three tests. Based on previously published research, we utilized the lowest quartile of the score as the cut-off value [22–24]. For the CERAD test, the cut-off value for low cognitive performance was 22. Regarding the Animal Fluency test, the cut-off point was 15, and for the DSST, the cut-off value was 42. Considering that age was a major risk factor for cognitive performance, the score was further categorized based on age (“60–69 years”, “70–79 years”, and “80+”) [23, 24]. Those who scored below the corresponding cut-off values were considered to constitute the low cognition group, and the others were considered to constitute the normal cognition group.

Based on age, sex, ethnicity, and serum creatinine (Scr) concentration, we used the Modification of Diet in Renal Disease (MDRD) equation to calculate the eGFR [25]. According to previously published guidelines, we categorized CKD stage as follows: no CKD, CKD stages 1–2 (eGFR ≥ 60 mL/min/1.73 m^2^ and uACR ≥ 30 mg/g); and CKD stages 3–5 (eGFR < 60 mL/min/1.73 m ^2^) [26].

### Covariates

Potential covariates included age (“60–69 years”, “70–79 years”, and “80+”), sex (“female” and “male”), ethnicity (“non-Hispanic White”, “non-Hispanic Black”, “other Hispanic”, “other/multiracial”, and “mexican American”), education attainment (“low education” and “high education”), family income(poverty income ratio, PIR), physical measures [body mass index (BMI) and waist circumference], smoking status (“current”, “former”, and “never”), alcohol drinking, comorbidities (hypertension and diabetes), and the uACR.

### Statistical analysis

The sample weights of the individuals in the mobile examination centers were taken into account according to the NHANES analytical guidelines [27]. The sample weight of this study was the half of the original 2-year sample weight since involved two cycles of continuous data were included. Continuous variables are represented as the weighted mean ± standard deviation, and weighted percentages are presented as the frequency for categorical variables. Associations of covariates between CKD stage and cognitive performance were assessed via the Wilcoxon rank-sum test for continuous variables and the chi-square test with Rao & Scott’s second-order correction for categorical variables. Following previous research, we used multivariate adjusted logistic regression models to quantify the association between CKD stage and each individual cognitive ability through the odds ratios (ORs) and 95% confidence intervals (CIs). Model 1, we controlled for age, sex, and ethnicity. Model 2 was adjusted for Model 1 covariates plus education attainment, PIR, BMI, waist circumference, alcohol drinking, smoking status, diabetes, and hypertension. We performed a subgroup analysis in order to examine the reliability of the results. The restricted cubic spline was used for the dose-response relationship in the logistic regression Model 2. All the data analyses were performed in R version 4.3.1. A two-tailed *p* value < 0.05 was regarded as statistically significance.

## Results

The baseline characteristics of the 2234 participants are summarized in Table 1. Participants with poor cognitive function may be older; be non-black; have lower educational attainment, PIR, alcohol drinking capacity, or eGFR; and have a greater incidence of albuminuria (uACR ≥ 30 mg/g) and CKD. According to the CERAD and Animal Fluency tests, people with low cognitive performance were more likely to be male. The prevalence of hypertension and diabetes in people identified with low cognitive performance according to the CERAD test and Animal Fluency test was significantly greater than that in people with normal cognitive performance. Moreover, people with low cognitive performance according to the DSST had a greater probability of being current or former smokers.

**Table 1:** Weighted baseline characteristics of participants from 2011 to 2014 NHANES

	CERAD test				DSST			Animal Fluency test		
**Characteristic**	**Overall**, *N* = 2234 (100%)^1^	**Low cognition**, *N* = 715 (26%)^1^	**Normal cognition**, *N* = 1519 (74%)^1^	***p *** **value** ^2^	**Low cognition**, *N* = 900 (26%)^1^	**Normal cognition***N* = 1334 (74%)^1^	***p *** **value** ^2^	**Low cognition**, *N* = 945 (32%)^1^	**Normal cognition**, *N* = 1289 (68%)^1^	***p *** **value** ^2^
**Age (years)**				**< 0.001**			**< 0.001**			**< 0.001**
60–69	1,144 (53%)	271 (32%)	873 (61%)		364 (33%)	780 (61%)		425 (38%)	719 (61%)	
70–79	614 (27%)	212 (33%)	402 (25%)		280 (35%)	334 (24%)		276 (34%)	338 (24%)	
80+	476 (20%)	232 (34%)	244 (14%)		256 (33%)	220 (15%)		244 (28%)	232 (16%)	
**Sex (female)**	1,142 (54%)	283 (44%)	859 (57%)	**< 0.001**	380 (48%)	762 (56%)	**0.010**	487 (54%)	655 (53%)	0.9
**Ethnicity**				**0.004**			**< 0.001**			**< 0.001**
Non-Hispanic White	1,117 (81%)	347 (77%)	770 (82%)		321 (65%)	796 (87%)		365 (70%)	752 (86%)	
Non-Hispanic Black	493 (7.4%)	155 (8.3%)	338 (7.1%)		274 (16%)	219 (4.5%)		271 (13%)	222 (4.8%)	
Other Hispanic	225 (3.5%)	90 (5.5%)	135 (2.8%)		135 (8.2%)	90 (1.8%)		114 (5.7%)	111 (2.5%)	
Other/multiracial	207 (4.8%)	51 (4.4%)	156 (4.9%)		62 (4.3%)	145 (5.0%)		112 (7.2%)	95 (3.7%)	
Mexican American	192 (3.2%)	72 (4.5%)	120 (2.8%)		108 (6.8%)	84 (2.0%)		83 (4.4%)	109 (2.7%)	
**Education attainment**				**< 0.001**			**< 0.001**			**< 0.001**
high education	1,183 (64%)	270 (47%)	913 (70%)		275 (37%)	908 (73%)		372 (46%)	811 (72%)	
low education	1,051 (36%)	445 (53%)	606 (30%)		625 (63%)	426 (27%)		573 (54%)	478 (28%)	
**PIR**	3.17 (1.58)	2.54 (1.51)	3.40 (1.54)	**< 0.001**	2.24 (1.43)	3.50 (1.49)	**< 0.001**	2.61 (1.52)	3.43 (1.53)	**< 0.001**
**BMI (kg/m** ^**2**^ **)**	29.0 (6.1)	28.6 (5.7)	29.1 (6.2)	0.3	28.6 (6.1)	29.1 (6.1)	0.2	29.0 (6.4)	29.0 (5.9)	0.7
**Waist circumference (cm)**	102 (15)	103 (14)	102 (15)	0.6	102 (15)	103 (15)	0.7	103 (15)	102 (14)	> 0.9
**Smoking status**				> 0.9			**0.004**			0.2
Current	287 (11%)	100 (11%)	187 (11%)		144 (14%)	143 (10%)		137 (14%)	150 (10%)	
Former	854 (39%)	276 (40%)	578 (39%)		354 (44%)	500 (38%)		350 (39%)	504 (39%)	
Never	1,093 (49%)	339 (48%)	754 (50%)		402 (42%)	691 (52%)		458 (47%)	635 (50%)	
**Alcohol drinking**	1,560 (74%)	495 (70%)	1,065 (76%)	**0.009**	586 (66%)	974 (77%)	**< 0.001**	618 (68%)	942 (77%)	**< 0.001**
**Hypertension**	1,373 (58%)	456 (64%)	917 (56%)	0.050	604 (66%)	769 (55%)	**< 0.001**	631 (64%)	742 (55%)	**< 0.001**
**Diabetes**	528 (19%)	189 (23%)	339 (18%)	0.058	261 (27%)	267 (17%)	**< 0.001**	262 (25%)	266 (17%)	**< 0.001**
**uACR**	46 (297)	62 (291)	40 (299)	**0.008**	97 (440)	28 (225)	**< 0.001**	79 (433)	31 (205)	**< 0.001**
**eGFR (ml/min/ 1.73m** ^**2**^ **)**	73 (19)	69 (21)	74 (18)	**< 0.001**	69 (23)	74 (18)	**0.001**	70 (22)	74 (17)	**0.001**
**CKDgroup**				**< 0.001**			**< 0.001**			**< 0.001**
No CKD	1,452 (67%)	408 (57%)	1,044 (71%)		500 (54%)	952 (72%)		550 (58%)	902 (72%)	
Stages 1–2	259 (9.1%)	98 (11%)	161 (8.5%)		137 (13%)	122 (7.6%)		123 (10%)	136 (8.5%)	
Stages 3–5	523 (24%)	209 (33%)	314 (20%)		263 (33%)	260 (20%)		272 (32%)	251 (20%)	

^1^Median ± standard deviation for continuous; n (%) for categorical

^2^Chi-squared test with Rao & Scott’s second-order correction

*p* < 0 0.05 is written in bold letters

Table 2 presents the associations between different stages of CKD and cognitive performance. According to the CERAD test and Animal Fluency test scores, there was a statistically significant association between CKD stages 3–5 and low cognitive performance. For the DSST score, each CKD stage was statistically associated with poor cognitive performance. After full adjustment for the potential confounders (Model 2), the multivariate adjusted OR for low cognitive performance according to the CERAD test score was 0.70 (95% CI: 0.51, 0.97). The Animal Fluency test score had a multivariate-adjusted OR (95% CI) of 0.64 (0.48, 0.85). The multivariate adjusted ORs of low cognitive performance according to DSST score were 0.60 (95% CI: 0.37, 0.97) and 0.60 (95% CI: 0.41, 0.88), respectively.

**Table 2:** Weighted OR (95% CIs) of stage of CKD for scores on the CERAD test, Animal Fluency test and DSST

Characteries	CERAD test		DSST		Animal Fluency test	
	OR (95% CI)	*p*	OR (95% CI)	*p*	OR (95% CI)	*p*
**Unadjusted**						
No CKD	ref	ref	ref	ref	ref	ref
Stages 1–2	0.64 (0.43, 0.93)	**0.022**	0.43 (0.28, 0.65)	**< 0.001**	0.66 (0.43,1.01)	0.053
Stages 3–5	0.50 (0.39, 0.65)	**< 0.001**	0.45 (0.34, 0.61)	**< 0.001**	0.49 (0.20,0.61)	**< 0.001**
**Model 1**						
No CKD	ref	ref	ref	ref	ref	ref
Stages 1–2	0.74 (0.50, 1.08)	0.12	0.50 (0.32, 0.78)	**0.004**	0.81 (0.56, 1.17)	0.2
Stages 3–5	0.63 (0.49, 0.83)	**0.002**	0.53 (0.37, 0.75)	**0.001**	0.57 (0.44, 0.75)	**< 0.001**
**Model 2**						
No CKD	ref	ref	ref	ref	ref	ref
Stages 1–2	0.84 (0.60, 1.18)	0.30	0.60 (0.37, 0.97)	**0.04**	0.97 (0.66, 1.43)	0.9
Stages 3–5	0.70 (0.51, 0.97)	**0.033**	0.60 (0.41,0.88)	**0.013**	0.64 (0.48, 0.85)	**0.005**

Calculated using multivariate-adjusted logistic regression; Model 1 adjusted for age, sex, ethnicity; Model 2, Model 1 additionally adjusted for education attainment, PIR, BMI, waist circumference, alcohol drinking, smoking status, diabetes, and hypertension

*p* < 0 0.05 is written in bold letters

Table 3 presents the association between the eGFR and cognitive performance. Compared to the lowest quartile of eGFR, there was statistically significant association between the eGFR and the score of three tests in unadjusted model. After adjustment for age, sex, and ethnicity, the DSST and Animal Fluency test scores were statistically significant associated with the eGFR. For the CERAD test score, we found a significant association between the quartile two of eGFR and low cognitive performance. According to the Model 2, compared with that of lowest quartile of eGFR, the multivariate adjusted OR of the quartile two was 1.53 (95% CI: 1.03, 2.27) for the CERAD test score. The multivariate-adjusted OR of the quartile three was 1.70 (95% CI: 1.06, 2.71) for the Animal Fluency test score. Besides, the multivariate adjusted ORs of low cognitive performance according to DSST score were 1.67 (95% CI: 1.10, 2.54) for the quartile two and 1.60 (95% CI: 1.07, 2.38) for the quartile three.

**Table 3:** Weighted OR (95% CIs) of eGFR for scores on the CERAD test, Animal Fluency test and DSST

Characteries	CERAD test		DSST		AnimalFluency test	
	OR (95% CI)	*p*	OR (95% CI)	*p*	OR (95% CI)	*p*
**Unadjusted**						
Q1	ref	ref	ref	ref	ref	ref
Q2	1.89 (1.37, 2.61)	**< 0.001**	2.22 (1.54, 3.20)	**< 0.001**	2.12 (1.52, 2.97)	**< 0.001**
Q3 Q4	1.82 (1.33, 2.49) 1.94 (1.36, 2.70)	**< 0.001** **< 0.001**	2.18 (1.52, 3.11) 1.55 (1.15, 2.09)	**< 0.001** **< 0.005**	2.01 (1.48, 2.74) 1.61 (1.26, 2.07)	**< 0.001** **< 0.001**
**Model 1**						
Q1	ref	ref	ref	ref	ref	ref
Q2	1.71 (1.19, 2.45)	**0.005**	2.05 (1.28, 3.29)	**0.004**	1.91 (1.31, 2.78)	**0.002**
Q3 Q4	1.34 (0.98, 1.83) 1.43 (1.00, 2.05)	0.065 0.05	1.70 (1.17, 2.47) 1.33 (0.90, 1.96)	**0.008** **0.027**	1.63 (1.13, 2.33) 1.42 (1.05, 1.93)	**0.01** **0.027**
**Model 2**						
Q1	ref	ref	ref	ref	ref	ref
Q2	1.53 (1.03, 2.27)	**0.037**	1.71 (0.99,2.94)	0.054	1.67 (1.10, 2.54)	**0.02**
Q3 Q4	1.33 (0.95, 1.85) 1.31 (0.83, 2.09)	0.09 0.2	1.70 (1.06,2.71) 1.18 (0.81, 1.72)	**0.03** 0.3	1.60 (1.07, 2.38) 1.29(0.93, 1.78)	**0.024** 0.12

eGFR (mL/min/1.73 m^2^): Q1(6.76–60.94), Q2 (60.95–73.23), Q3 (73.24–86.91), Q4 (86.92-157.03); calculated using multivariate-adjusted logistic regression; Model 1 adjusted for age, sex, ethnicity; Model 2, model 1 additionally adjusted for education attainment, PIR, BMI, waist circumference, alcohol drinking, smoking status, diabetes, and hypertension

*p* < 0 0.05 is written in bold letters

Table 4 shows the associations between the eGFR and cognitive performance as covariates. We performed a subgroup analysis which stratified by age, sex, hypertension, diabetes, smoking status, and education attainment. The results revealed that no significant dependence of the relationship between the eGFR and CERAD test score or Animal Fluency test score and age, sex, hypertension, diabetes, or smoke status (*p* > 0.05), expect for the education attainment (low education) of the participant taking the Animal Fluency test (OR = 1, 95% CI [0.99, 1.02], *p* = 0.045). Similarly, other statistically significant associations were not found between the eGFR and DSST score except for the female sex (OR = 1.01, 95% CI [1.0, 1.02], *p* = 0.006), current smoking status (OR = 1.02, 95% CI [1.0, 1.04], *p* = 0.016), or low education attainment (OR = 1.0, 95% CI [0.99, 1.02], *p* = 0.009).

**Table 4:** Subgroup analysis

Subgroup	CERADtest		DSST		Animal Fluency test	
	OR (95% CI)	*p*	OR (95% CI)	*p*	OR (95% CI)	*p*
**Age (years)**						
60–69	1.01 (1.0, 1.02)	0.11	1.0 (1.0, 1.03)	0.072	1.01 (1.0, 1.02)	0.2
70–79	1.01 (1.0, 1.02)	0.4	1.0 (0.99, 1.01)	> 0.9	1.0 (0.99, 1.01)	0.6
80+	1.0 (0.98, 1.01)	0.5	1.01 (1.0, 1.02)	0.11	1.01(1.0, 1.03)	0.081
**Gender**						
Female	1.0 (0.99, 1.01)	> 0.9	1.01 (1.0, 1.02)	**0.006**	1.01 (1.0, 1.02)	0.072
Male	1.01 (1.0, 1.02)	0.2	1.0 (0.99, 1.01)	> 0.9	1.0 (0.99, 1.01)	> 0.9
**Hypertension**						
Yes	1.0 (0.99, 1.01)	0.6	1.01 (1.0, 1.01)	0.14	1.01 (1.0, 1.02)	0.2
No	1.01 (1.0, 1.02)	0.1	1.01 (0.99, 1.02)	0.3	1.0 (0.98, 1.01)	0.5
**Diabetes**						
Yes	1.0 (0.99, 1.01)	0.5	1.01 (0.99, 1.03)	0.4	1.01 (1.0,1.03)	0.089
No	1.0 (1.0, 1.01)	0.3	1.0 (1.0, 1.02)	0.072	1.0 (0.99,1.01)	0.80
**Smoke status**						
Current	1.0 (0.99, 1.02)	0.7	1.02 (1.0, 1.04)	**0.016**	1.0 (0.99, 1.02)	0.8
Former	1.0 (0.99, 1.02)	0.4	1.0 (0.99, 1.02)	0.2	1.01 (1.0, 1.02)	0.2
Never	1.0 (0.99, 1.01)	0.7	1.0 (0.99, 1.02)	0.6	1.0 (0.99, 1.02)	0.5
**Education attainment**						
High education	1.0 (0.99, 1.01)	0.4	1.0 (0.99, 1.01)	0.6	1.0 (0.99, 1.01)	0.9
Low education	1.0 (0.99, 1.01)	0.2	1.0 (0.99, 1.02)	**0.009**	1.0 (0.99, 1.02)	**0.045**

All presented covariates were fully adjusted (as Model 2)

*p* < 0 0.05 is written in bold letters

**Fig. 2:**
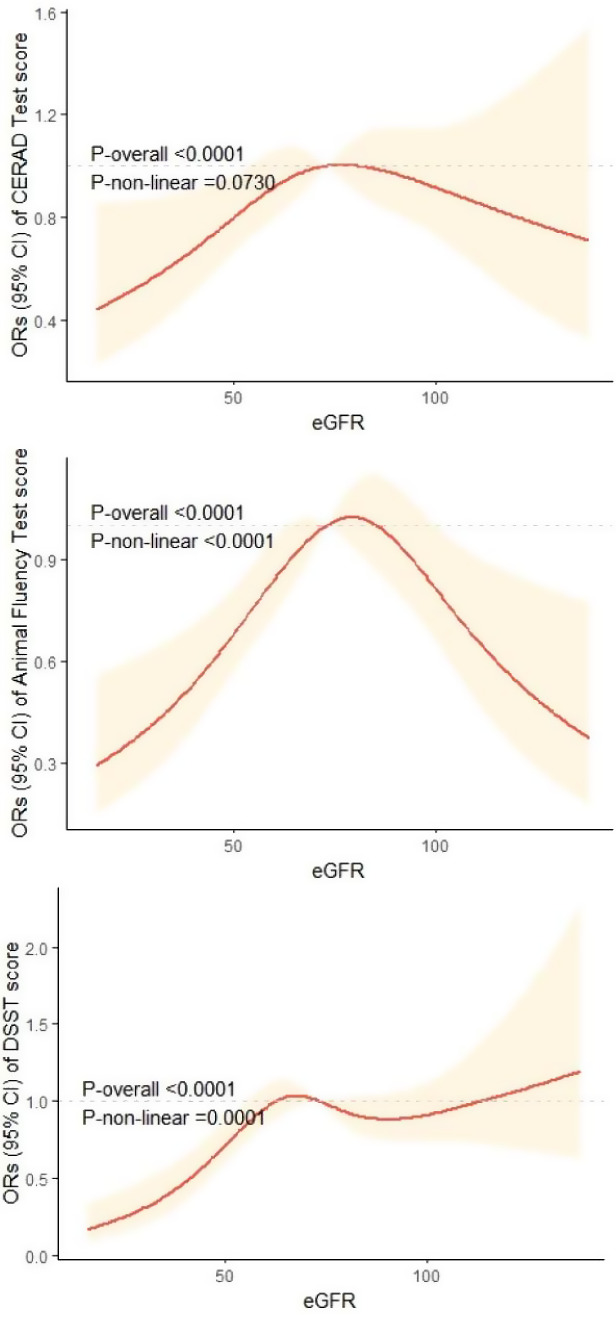
Dose–response relationship between eGFR and CERAD test score, DSST score, and Animal Fluency test score

Figure 2 depicts the results of the restricted cubic spline analyses. The prevalence of low cognitive function increased with decreasing eGFR. After full adjustment for multiple confounding factors, an inverted U-shaped dose-response relationship existed between the eGFR and the OR of the Animal Fluency test score. We also found an S-shaped relationship between the eGFR and the OR of the DSST score. Both the CERAD test score (*p* nonlinearity < 0.0001) and the Animal Fluency score test (*p* nonlinearity = 0.0001) exhibited a nonlinear dose–response relationship with eGFR. However, a linear relationship was shown between the eGFR and the OR of CERAD test score (*p* nonlinearity = 0.0730).

## Discussion

This study included 2234 participants (≥ 60 years old) from the NHANES 2011–2014. The results showed that participants with worse kidney function were more likely to have cognitive impairment than those with normal kidney function. In addition, these results were not affected by confounders known to confound the association between CKD and cognitive performance. According to the sensitivity analyses, there was an association between the eGFR and DSST score in females, former and current smokers, and individuals with lower education attainment. A lower education attainment may influence Animal Fluency test scores. Moreover, we found that there was a nonlinear relationship between the eGFR and the Animal Fluency test score and DSST score, but the eGFR and the CERAD test score showed a linear relationship.

Our results were partially consistent with those of previously published research. For example, a study of stroke risk factors indicated that poor kidney function was linked to an increased incidence of cognitive impairment in American adults [28]. In addition, a multicenter study by Sarnak et al. [29] demonstrated that patients of hemodialysis had greater prevalence of poor cognitive function, which mainly influences executive function. Similarly, Kalirao et al. [30] suggested that more peritoneal dialysis patients have serious cognitive impairment than hemodialysis patients. Overall, although we used different tests to evaluate cognitive performance and different formulas were used to assess kidney function, CKD may be a crucial marker of cognitive impairment in adults. Additionally, dialysis modality is not the only factor contributing to the pathogenesis of CKD-related cognitive impairment. The initiation of cognitive performance assessment therapy before and after dialysis begins may benefit these patients.

In addition, there was a decline in cognitive performance in the early stages of CKD (stages 1–2) in our study results, which has also been replicated in previously published studies. In 2004, Seliger et al. [31] showed that moderate kidney injury is closely related to a high risk of incident dementia among healthy individuals. Similarly, Hailpern et al. [32] also indicated that among a large nationally representative sample of adults, moderate CKD was strongly linked to poorer visual attention, learning, and concentration. However, these results were mainly reflected in the cognitive performance of the participants tested using the DSST. The lack of standardization has been presented as a problem in cognitive performance screening. Clinicians frequently depend on subjective, self-perceived screening rather than objective, comprehensive examinations. Given that these study results are parallel to current clinical practice, nephrologists should use objective and comprehensive tests to detect cognitive impairment associated with decreased kidney function when available. Of course, it is also worth to further discussed whether cognitive function decline occurs in the early or moderate stages of CKD.

Currently, studies have proven the association between CKD and cognitive impairment in the elderly individuals with comorbid conditions, as well as in menopausal women with coronary artery disease [33]. In the basis of our study results, we confirmed that CKD stages 3–5 were significantly associated with decreased cognitive function in the American adults. However, it is estimated that the incidence of CKD in young individuals is sharply increasing [34]. Ruebner et al. [35] indicated that a lower eGFR in adolescents and young adults was associated with poorer neurocognitive performance, particularly in attention, memory, and inhibitory control. Therefore, cognitive impairment is evident across the CKD patients, possibly independent of age-related changes. Although this study focused on people older than 60 years, there is no doubt that CKD patients need more attention given their cognitive performance. The long-term prognosis and quality of life should be improved, and the incidence of cognitive impairment should be decreased or delayed as much as possible.

We classified the eGFR into four categories to further explore the relationship between the eGFR and cognitive performance. The results showed that low and moderate eGFR are closely linked to poor cognitive performance in elderly individuals. However, a study including 4095 community-dwelling individuals aged 35 to 82 years revealed that the eGFR was almost irrelevant to cognitive performance [36]. Similarly, Martens et al. [37] reported that the eGFR was not related to cognitive performance in the entire study population aged 40 to 75 years. These findings contradicted most of the study results mentioned in previous articles and this study [28, 32]. The difference in the results could be due to the inclusion of a number of young community-based populations with normal or moderately impaired eGFRs [36]. Many researchers have concentrated on older or high-risk groups and have included more people with low eGFRs [28, 32]. In addition, the confounding effect of the model affects the outcome, especially for cardiovascular risk and diabetes. After all, these studies have been shown a link between CKD and all-cause and cardiovascular mortality and diabetes [38, 39].

Moreover, we discovered a significant nonlinear relationship between the eGFR and the incidence of cognitive impairment, especially according to the Animal Fluency test score and DSST scores. Notably, studies ignoring this nonlinear relationship may underrate the real connection between kidney disease and cognitive impairment. Several studies have used glomerular biomarkers of kidney health to determine the association between kidney function and cognitive performance in individuals with a normal eGFR individuals [40, 41]. However, Miller et al. [42] indicated that urine biomarkers of tubule injury, fibrosis, and proximal tubule resorptive capacity are variably connected to the worsening cognitive performance, independent of glomerular markers of kidney health. Hence, identifying kidney function biomarkers in healthy individuals may be an excellent method to further evaluate the associations between kidney function and cognitive performance.

Our research has several strengths. The major strengths of this study were the use of a large-scale, nationally representative cohort of healthy, ethnically diverse elderly individuals in the U.S. In addition, the NHANES database was used for rigorous methods and quality control. Moreover, various ranges of potential confounders were accounted for. We also explored the dose–response relationship between the eGFR and cognitive performance. Nonetheless, our study also has several limitations. The association between CKD and cognitive function cannot be considered causal. In addition, three tests did not cover all domains of cognitive performance. Finally, the findings may have racial limitations and cannot be applied to people with other specific diseases.

## Conclusion

CKD, especially CKD stages 3–5, was significantly associated with low cognitive performance in executive function, learning, processing speed, concentration, and working memory ability. These findings added evidence to the existing study on the aged patients with CKD, and may provide crucial insights into the development of cognitive impairment. In the future, well conducted longitudinal studies of multiple cognitive functions in adults at different CKD stages are needed.

## Data Availability

All the data are available on the NHANES website: https://wwwn.cdc.gov/nchs/nhanes/default.aspx.

## Abbreviations

BMI: body mass index
CKD: chronic kidney disease
CERAD: Consortium to Establish a Registry for Alzheimer's Disease
CI: confidence interval
DSST: Digit Symbol Substitution Test
eGFR: estimated glomerular filtration rate
NHANES: National Health and Nutrition Examination Survey
OR: odds of ratio
PIR: poverty income ratio
ref: reference
Scr: serum creatinine
uACR: urinary albumin to creatinine ratio
